# Purulent Pericarditis: Treating a Medically-Complex Patient With Intrapericardial Antibiotics

**DOI:** 10.1016/j.jaccas.2025.105152

**Published:** 2025-09-24

**Authors:** Meryem Guler, Sudheshna Vemula, Matthew Hart, Josh Wiener, Darius Farzad, Mark DeCaro, Gregary D. Marhefka, Devin Weber, John Entwistle, Jacqueline Urtecho

**Affiliations:** a Sidney Kimmel Medical College at Thomas Jefferson University, Philadelphia, Pennsylvania, USA; b Department of Neurology and Neurological Surgery, Thomas Jefferson University Hospital, Philadelphia, Pennsylvania, USA; c Division of Cardiology, Department of Medicine, Thomas Jefferson University Hospital, Philadelphia, Pennsylvania, USA; d Division of Infectious Diseases, Department of Medicine, Thomas Jefferson University Hospital, Philadelphia, Pennsylvania, USA; e Department of Cardiac Surgery, Thomas Jefferson University Hospital, Philadelphia, Pennsylvania, USA

**Keywords:** bacterial pericarditis, intrapericardial antibiotics, intrapericardial infusion, pericardial drain, purulent pericarditis

## Abstract

**Objective:**

Purulent pericarditis is a rare, lethal complication of a bacterial pericardial infection. This case report outlines the protocol for instilling intrapericardial antibiotics for methicillin-sensitive *Staphylococcus aureus* (MSSA) pericarditis without fibrinolytics in a 51-year-old man.

**Key Steps:**

An appropriately sized percutaneous pericardial drain was placed. Then, a 3-day course of intrapericardial infusions of 50 mL vancomycin was begun. Drainage of all accessible fluid was performed before each infusion. The daily infusions each had an 8-hour dwell time. The prior drainage protocol continued after each infusion.

**Potential Pitfalls:**

Strict dosage and infusion length management are required to prevent direct toxicity. Potential complications include local inflammatory responses and constrictive pericarditis.

**Take-Home Messages:**

The use of simultaneous intravenous and intrapericardial antibiotics were used for treating MSSA purulent pericarditis without using intrapericardial fibrinolytics in an adult. This was noted to favorably improve the purulent output and relative safety without untoward complications.

## Visual Summary

**Figure:**
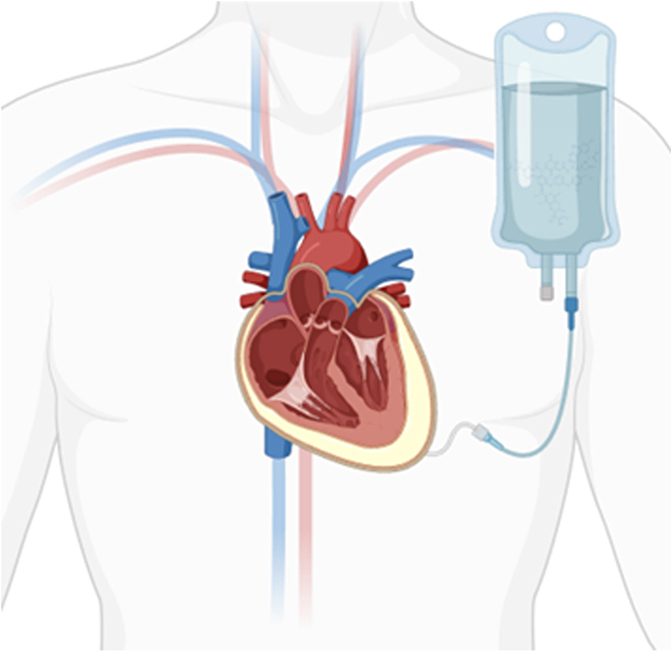

**Visual Summary:**
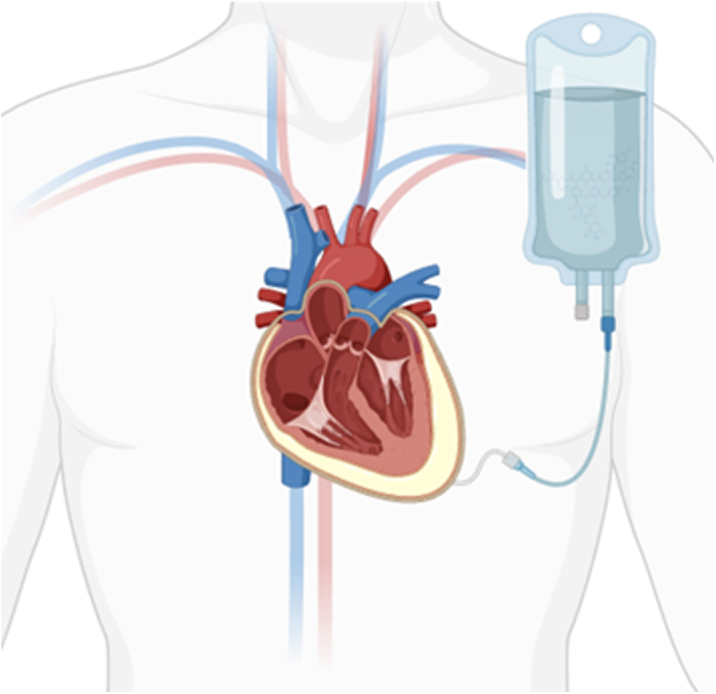
Intrapericardial Infusion and Dwelling of Antibiotics Via Pericardial Drain

Historically, the incidence of bacterial pericarditis, and specifically purulent pericarditis, has dramatically declined since the 1940s with the introduction of antibiotics and later vaccines. In the modern era, the presence of purulent pericarditis is quite rare, with a reported incidence of approximately 0.6% among a cohort of 500 patients diagnosed with acute pericarditis.^1^ However, it carries a significant mortality rate of approximately 40% with treatment and 100% without, making its rapid diagnosis and management paramount to achieve positive outcomes.^2^^,^^3^

**Take-Home Messages**

• We outline the use of simultaneous intravenous and intrapericardial antibiotics for treating MSSA purulent pericarditis without using intrapericardial fibrinolytics in an adult.
• This was noted to favorably improve the purulent output and relative safety without untoward complications.

Given the severity of this condition and the high associated mortality rate, management requires immediate and intensive treatment. Broad-spectrum antibiotics are often started intravenously while drainage options are weighed, ranging from conservative to more invasive. These include percutaneous pericardiocentesis, subxiphoid pericardiotomy, video-assisted thoracic surgery, or sternotomy and pericardiectomy.^2^^,^^4^ Open drainage options are currently the preferred method of intervention. Still, there have been reports of intrapericardial infusions of fibrinolytics such as streptokinase to break down collections, with no recurrence of purulent fluid.^5^, ^6^, ^7^ Extra caution must be taken with streptokinase in the pericardium owing to its association with greater bleed risk. Although intrapericardial antibiotics have been discussed in the literature, including cases with concomitant intrapericardial antibiotics and fibrinolytics, limited reports exist on this method.^4^^,^^8^

In this case report, we discuss using simultaneous intrapericardial and systemic antibiotics for a medically complex patient with purulent bacterial pericarditis, without using fibrinolytics.

## Case Summary

A 51-year-old man presented to an outside hospital with complaints of shortness of breath after his last hemodialysis session 1 week prior. His past medical history was notable for end-stage renal disease on regular hemodialysis, right nephrectomy, hyperlipidemia, hypertension, pulmonary hypertension, obstructive sleep apnea, and a well-healed below-the-knee amputation of the left leg. He was found to have a severe metabolic acidosis (pH: 7.0, CO_2_: 41, bicarbonate: 10, lactate: 14), fluid overload, and septic shock secondary to methicillin-sensitive *Staphylococcus aureus* (MSSA) bacteremia. Despite emergent hemodialysis, bilevel positive airway pressure, intravenous sodium bicarbonate, intravenous cefazolin, and vasopressors, he required intubation the next day for worsening respiratory distress.

**Figure 1:**
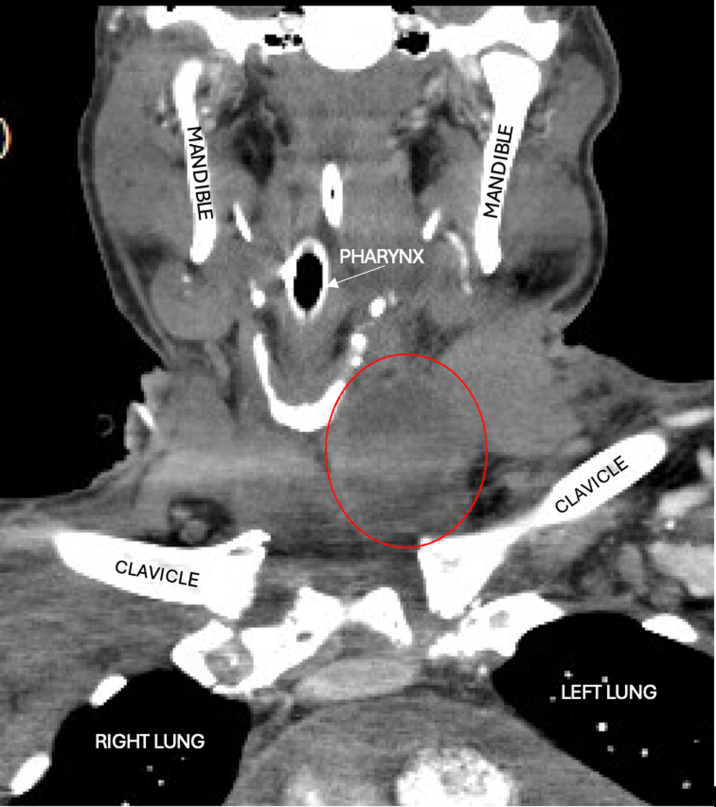
Computed Tomography of the Left Neck With Contrast Computed tomography coronal view of the chest/neck with contrast showing a rim-enhancing fluid collection (red circle) in the left neck.

**Figure 2:**
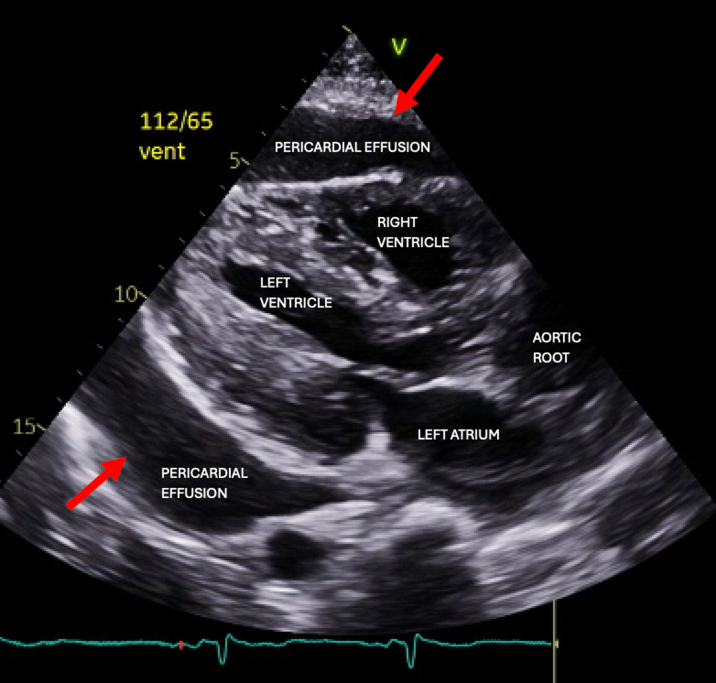
Pretreatment Echocardiogram Transthoracic echocardiogram parasternal long axis view showing moderate pericardial effusion (red arrows) without tamponade.

**Figure 3:**
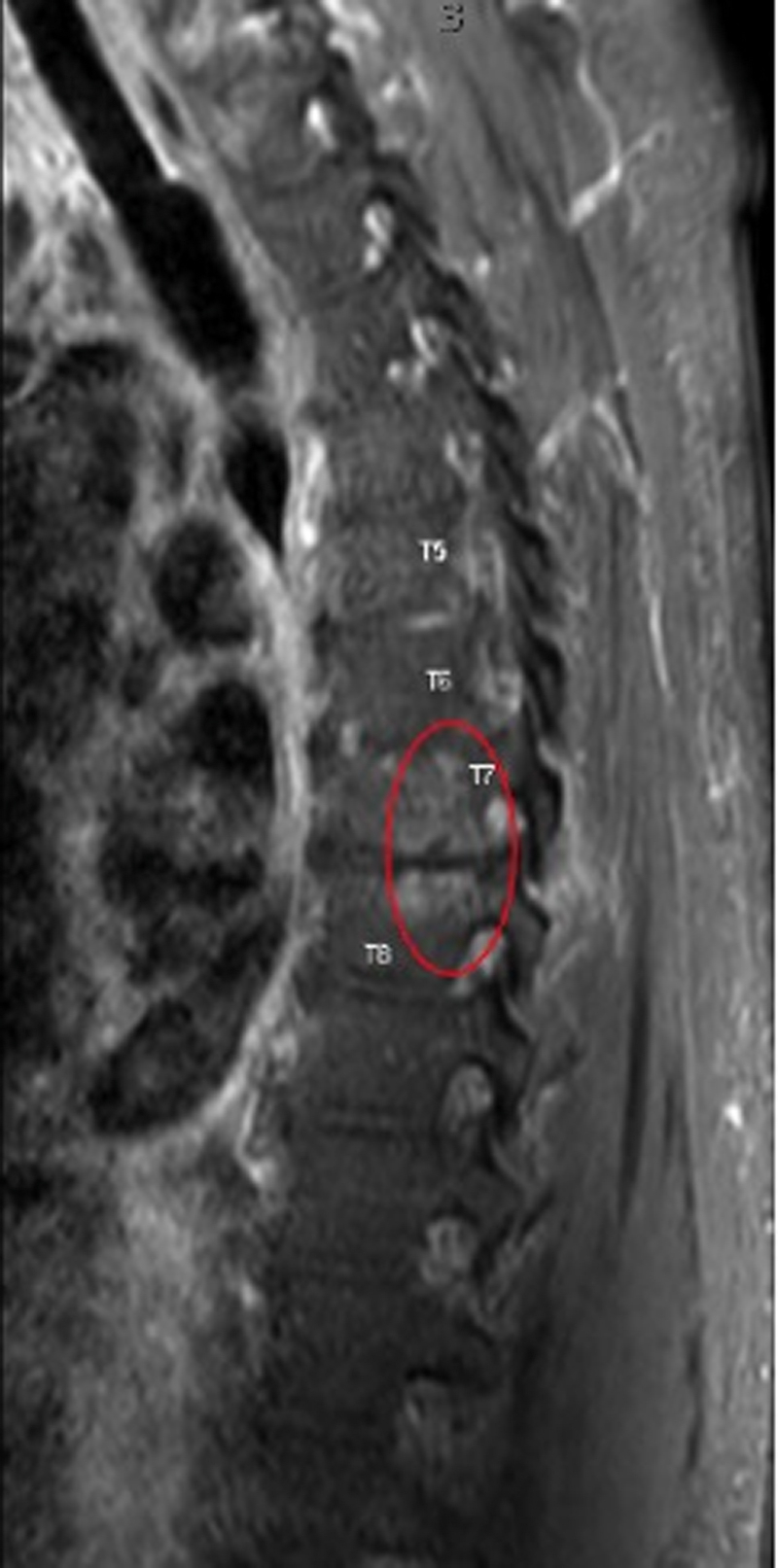
MRI of Thoracic Spine With Osteomyelitis Sagittal postcontrast T1-weighted MRI of the thoracic spine demonstrates enhancement in the T5-T8 vertebral bodies (red ellipse) consistent with osteomyelitis. MRI = magnetic resonance imaging.

**Figure 4:**
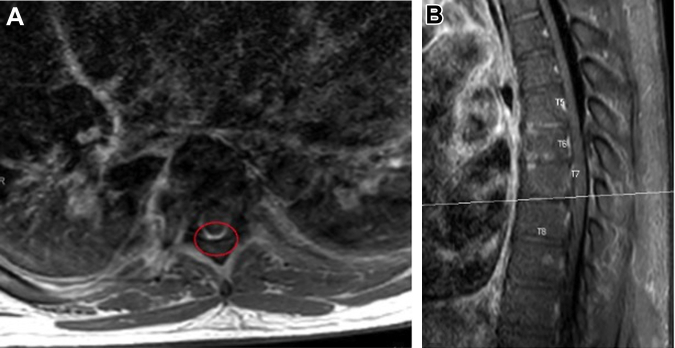
MRI of Thoracic Spine With Epidural Abscess (A) Axial postcontrast T1-weighted MRI at T7 and T8 with posterior epidural enhancement consistent with early epidural abscess formation (red ellipse). (B) Correlating sagittal postcontrast T1-weighted MRI with evidence of spinal epidural abscess formation at T7 and T8. MRI = magnetic resonance imaging.

Computed tomography showed a moderate pericardial effusion, ascites, interstitial pulmonary edema, and a rim-enhancing fluid collection in the left neck (Figure 1). The left neck abscess was aspirated, and cultures grew MSSA. Owing to persistently positive blood cultures, a transthoracic echocardiogram (TTE) and magnetic resonance imaging (MRI) of the entire spine were conducted to evaluate for a source of infection. TTE showed a medium- to large-sized pericardial effusion (Figure 2) without tamponade, normal biventricular function, mildly thickened valves without significant regurgitation, and no obvious vegetations. MRI spine with and without contrast exhibited discitis and osteomyelitis from C5 to C7 and T5 to T8. The latter revealed the spread of infection into the posterior thoracic epidural space, concerning for a developing abscess (Figures 3 and 4). The patient's neurological examination was negative for focal motor deficits but he exhibited mild confusion and drowsiness, likely secondary to infectious encephalopathy. He was transferred to our institution for neurosurgical and otolaryngological evaluation and treatment.

**Figure 5:**
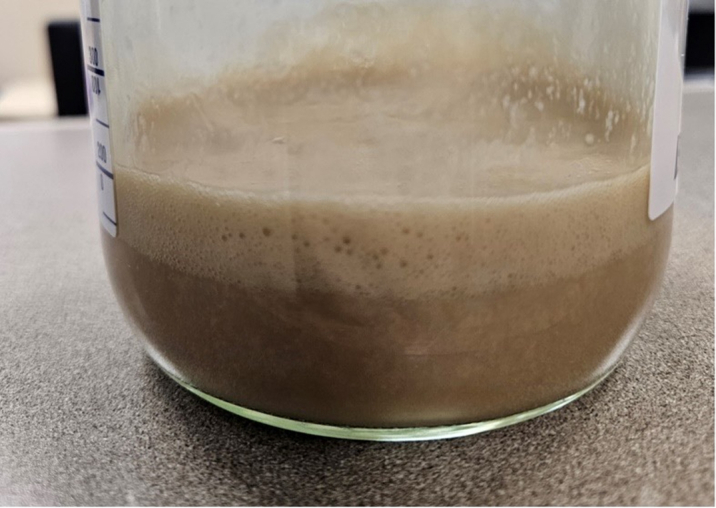
Pericardial Drainage Initial appearance of the tan-colored fluid aspirated from the pericardial space, totaling 1.35 L in the first 24 hours of drain placement.

The patient was admitted to our institution's neurocritical care unit for surgical intervention. On arrival, he was awake and alert, and was able to follow simple commands once sedation was withheld. His motor examination at that time was consistent with his previous baseline. Notably, the examination did not reveal any hyper-reflexia, accurately corresponding to the absence of severe spinal cord compression. Subsequently, a repeat TTE demonstrated enlargement of the complex effusion without tamponade physiology. This prompted the cardiology team to proceed with pericardiocentesis and drain placement to provide stability before surgical drainage of the neck and spinal epidural abscesses. Initial pericardial drainage was noted to be 250 mL of thick, tan-colored fluid (Figure 5), which brought up concerns of either infectious etiology or a chylous component. Fluid analysis showed protein 4.6 g/dL, lactate dehydrogenase 51,200 IU/L, glucose <2 mg/dL, many white blood cells, and moderate gram-positive cocci. Cytology was negative. The nursing team drained fluid per our institutional protocol, every 2 hours until <100 mL, then 4 hours until <100 mL, then every 8 hours. After each drainage, 2 mL of sterile saline was returned to the patient, and the 3-way stopcock was turned off.

**Figure 6:**
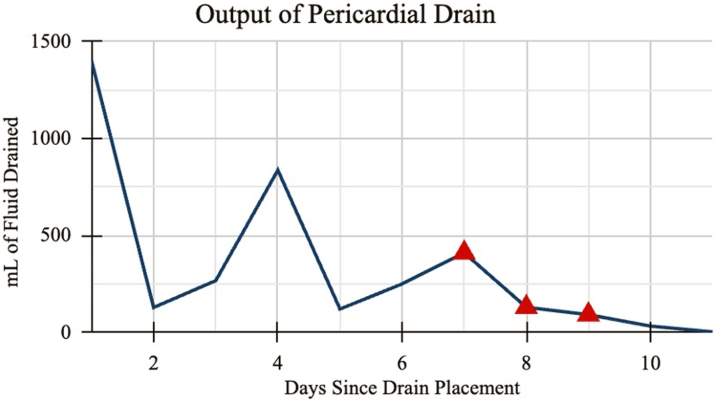
Graph of Pericardial Drainage Amount of pericardial fluid drainage (in milliliters) since drain placement. Red triangles indicate the days when intrapericardial vancomycin was infused. Drainage totals include amounts instilled into the pericardium.

Through the pericardial drain, the team extracted purulent fluid with an output of 1.35 L in the first 24 hours. In the following days, the daily production of this drain fluctuated, ranging from 121 to 836 mL (Figure 6). Cultures of the fluid grew MSSA, per his known MSSA bacteremia.

The cardiac surgery team was consulted for possible surgical intervention with either a washout of the pericardial space or with placement of a larger pericardial drain. The team felt that the patient was at high risk for postoperative complications. They agreed to continue intravenous antibiotics with regular pericardial drainage and to consider intrapericardial antibiotics and streptokinase, with control of infectious sources before considering a cardiac surgical approach.

A third TTE was performed after 7 days of renally dosed vancomycin. The effusion was smaller and loculated. An interdisciplinary team formed by the neurocritical care team included cardiology, cardiac surgery, infectious disease, and pharmacology. Consensus was to proceed with intrapericardial vancomycin infusion. Three doses of daily intrapericardial vancomycin infusions were started 7 days after the pericardial drain placement. On the first day of intrapericardial antibiotic infusion, the drain output was 410 mL, decreasing to 130 mL and 92 mL on days 2 and 3, respectively (Figure 5).

**Figure 7:**
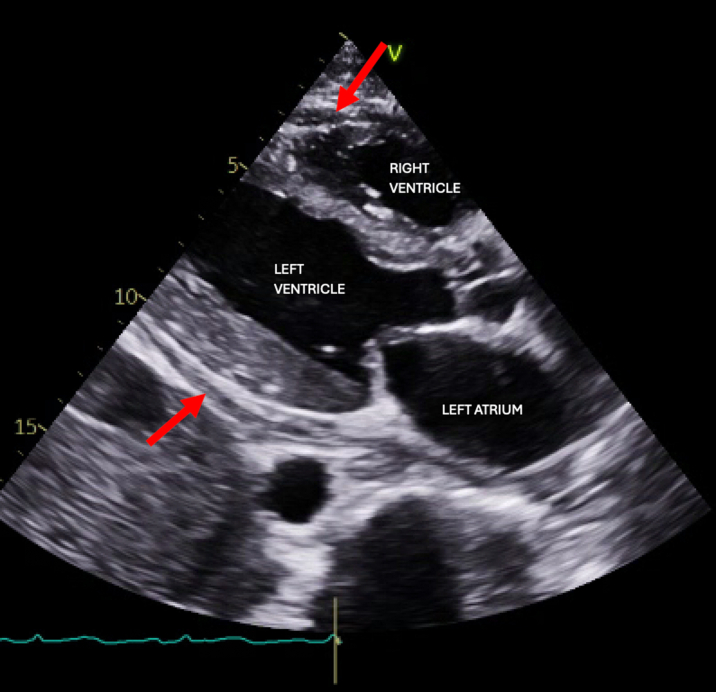
Post-treatment Echocardiogram Transthoracic echocardiogram parasternal long-axis view showing trivial, organized pericardial effusion (red arrows) 14 days after original pericardial drain placement.

The pericardial drain was removed on day 11 after the drainage was negligible. A fourth TTE on day 14 showed marked improvement, with a trivial organized posterior collection remaining (Figure 7). A final TTE on day 19 showed no increase in the known posterior collection. Follow-up for this patient was limited owing to his being discharged and transferred back to his home state once stabilized. He completed 1 month of intravenous cefazolin treatment as an outpatient and continues to receive regular hemodialysis sessions. To date, he reports chronic fatigue related to his end-stage renal disease, but no symptoms of cardiac failure or tamponade.

## Procedural Steps

The protocol used for instilling simultaneous intravenous and intrapericardial antibiotics in this patient was as follows:

1. Percutaneous pericardial drain placement was performed using a sterile technique with echocardiogram guidance and agitated saline injection confirmation, local lidocaine 1% infiltration, a micropuncture needle and catheter, J-wire, and a 6-F drain, which was upsized to an 8-F drain to facilitate drainage in this case.
2. Three days of intrapericardial vancomycin started on day 7 of pericardial fluid drainage. The inpatient pharmacology team calculated the dose of intrapericardial vancomycin infusion to be 50 mL of 1,000 mg vancomycin in 0.9% normal saline.
3. Daily intravenous cefazolin 1,000 mg doses were continued throughout the 3-day course of intrapericardial infusions.
4. Before each infusion, an empty syringe was applied to the 3-way stopcock between the pericardial drain and the drainage bag, using negative pressure, and any accessible fluid in the pericardial space was drained.
5. 50 mL of vancomycin was then placed into the pericardial space, and the 3-way stopcock was turned off for 8 hours each day of the antibiotic infusion.
6. After the 8-hour dwell time, an empty syringe was applied to the 3-way stopcock, using negative pressure, and any accessible fluid in the pericardial space was drained every 8 hours.
7. After the 3-day course of antibiotic infusions, the pericardial drain was drained every 8 hours per our institutional protocol. The drain was removed once the drainage dropped below 25 mL in a 24-hour period.

## Potential Pitfalls

Before administering intrapericardial vancomycin infusions, the interdisciplinary team involved in this patient's care considered potential pitfalls and complications that could result from this treatment method.

The first was overall poor efficacy due to poor penetration from inadequate concentrations of antibiotics or inaccessibility due to potential loculations within the pericardium. The simultaneous use of intrapericardial streptokinase or saline washouts can theoretically be used to mitigate the latter of these causes. Still, it was ultimately determined that the degree of loculations was minimal, and the team did not want to provoke intrapericardial bleeding, so streptokinase was not administered.

A bactericidal antibiotic such as vancomycin in the pericardial space could lead to a local inflammatory reaction in the pericardium or myocardium. If pericardial irritation occurs from infusions, this could ultimately lead to the downstream development of constrictive pericarditis. Additionally, incomplete eradication of the bacteria after completion of the antibiotic course can also lead to treatment resistance and recurrence.

## Conclusions

Few reports exist on intrapericardial antibiotics, perhaps owing to the efficacy of existing surgical treatment methods. However, when patients are deemed at high risk for a sternotomy and pericardial stripping, alternatives to treatment must be considered. This case highlights the benefit of multidisciplinary teamwork. It outlines the efficacy and safety of a complementary, direct infusion of antibiotics into the pericardial space in a medically complex patient with purulent pericarditis deemed at high risk for definitive surgery. Based on the results seen within this case and a few other case reports, further research into the effectiveness of this treatment method is warranted.

## Funding Support and Author Disclosures

The authors have reported that they have no relationships relevant to the contents of this paper to disclose.
